# Evaluation of microhardness, sorption, solubility, and color stability of bulk fill resins: A comparative study

**DOI:** 10.4317/jced.57599

**Published:** 2020-11-01

**Authors:** Luís-Felipe Espíndola-Castro, Márcia-de Almeida Durão, Tacyandra-Victória-Gomes Pereira, Ayanne-Karine-de Barros Cordeiro, Gabriela-Queiroz-de Melo Monteiro

**Affiliations:** 1 DDS, MSc, Dental School, Universidade de Pernambuco, Camaragibe Pernambuco, Brazil; 2 DDS, MSc, PhD, Dental School, Universidade de Pernambuco, Camaragibe Pernambuco, Brazil; 3 DDS student, Dental School, Universidade de Pernambuco, Camaragibe Pernambuco, Brazil

## Abstract

**Background:**

Due to the increasing popularity of bulk fill resins, there is a concern that their components can be leached; this is because these are inserted in a single 4-5 mm increment. This in vitro study evaluated the microhardness, sorption, solubility, and color stability of three restorative bulk fill resins, namely: Filtek Bulk Fill (FBF), Tetric N-Ceram Bulk Fill (TNC), and Opus Bulk Fill (OBF).

**Material and Methods:**

Cylindrical samples were fabricated to be 15 mm in diameter and 1 mm thick (n = 10). For the microhardness test, three random indentations were formulated on the samples using a micro-durometer with a load of 300 gf for 15 s. Sorption and solubility were then evaluated (ISO 4049: 2009). Color stability was analyzed with a digital spectrophotometer three times (initially, after 24 h, and after 7 d) during immersion in coffee and distilled water (control). The Shapiro-Wilk test was applied to analyze normality. The Mann-Whitney and Kruskal-Wallis tests were used to compare the groups and the immersion solution, with a significance level of 5%.

**Results:**

There were a significant difference in microhardness (*p*<0.001), with the FBF group showing a higher value compared to the other groups (56.38). The highest average of sorption scores was observed in the OBF group (16.9 µg / mm3), followed by FBF (16.8 µg / mm3) and TNC (11.3 µg / mm3). Solubility was lowest in the OBF group (-2.83 µg / mm3), with a significant difference (*p* = 0.031). There was also a significant difference after 24 h in the mean ∆E score of all groups (*p*<0.005). After one week of immersion, the group that pigmented most was OBF (*p* = 0.008).

**Conclusions:**

The three bulk fill resins had acceptable hardness, sorption, and solubility values. However, all groups showed a high pigmentation rate after 7 d of immersion in coffee.

** Key words:**Bulk fill, color stability, composite resins, microhardness, solubility, sorption.

## Introduction

Bulk fill composite resins were developed as a restorative alternative for posterior teeth to simplify the technique, minimize chances of failure, and reduce clinical time (1). The technology used for these materials allows them to be inserted in a single increment of up to 4 or 5 mm thickness (2,3). Randomized clinical trials show that bulk fill resins are clinically acceptable and have low annual failure rates (3). However, mechanical, optical, and biological properties such as hardness, color stability, sorption, and solubility could limit the use of these materials (4).

Different variables affect the longevity of composite resin restorations, including surface quality (5). Limited depth of cure and insufficient monomer conversion could result in inadequate microhardness (6,7). Composite resin restorations are also exposed to an aggressive oral environment with foods of varying temperatures and pigment consistencies (8). This can cause superficial changes in a short time by interfering with the mechanical and aesthetic properties of composite resins (8).

Staining of composite resins is multifactorial and may have an intrinsic etiology (degradation of the material due to its components), which is directly influenced by adequate light curing (9,10). In addition, composite resins can also undergo extrinsic staining due to the sorption of food coloring, medication, and nicotine, which leads to the production of an increased amount of mass (8,9,11). Color stability can be compromised based on the immersion solution, its pH, frequency, and contact time with the material; meanwhile, surface roughness also directly interferes with the intensity and speed of staining (12,13).

Water sorption generates an external movement of residual monomers and ions, which increases solubility (14). Sorption and solubility can be considered as precursors of several chemical and physical processes that facilitate the damage caused to the structure of the polymeric material, which can compromise its clinical effectiveness (14).

Thus, the aim of this study was to evaluate and compare the microhardness, sorption, solubility, and color stability of three restorative bulk fill resins at three different times, which were immersed in coffee and distilled water (control). The null hypotheses were established by the fact that between the different bulk fill resins, there is no difference in (1) microhardness, (2) color stability, (3) sorption, and (4) solubility.

## Material and Methods

-Materials

Three bulk fill restorative resins were evaluated in the present study and can be found in Table 1.

**Table 1:**
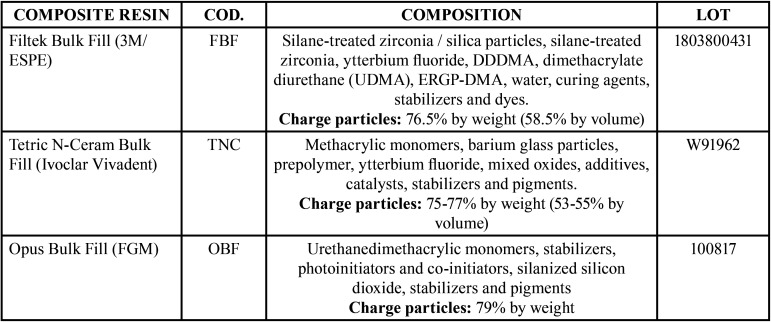
Materials tested and composition.

-Confection of the samples

To evaluate the color stability, microhardness, sorption, and solubility of the materials, 30 cylindrical specimens (10 per group) were fabricated in a bipartite metallic mold. The cylinders were each 15 mm in diameter and 1 mm thick. This standardization follows the ISO 4049: 2009 (15) standard for assessing sorption and solubility.

A metallic mold was placed on a glass blade and filled with resins. Another glass blade was placed on top and put under pressure using a glass plate (500g) for 20 s, and light curing (Radii, SDI, Victoria, Australia) was performed on both sides for 40 s at a power of 1.200 mW / cm2.

The dimensions of the specimens were standardized using sandpaper (#600, #1.000, and #1.500) and measured with a digital caliper (MDC-25 M, Mitutoyo, Tokyo, Japan) with an accuracy of ± 0.01 mm. Impurities were removed using an ultrasonic tub (Cristófoli, Paraná, Brazil) for 10 min, and the specimens were dried with compressed air.

-Microhardness

A digital micro-durometer (Insize, Model ISH-D120, São Paulo, Brazil) was used for the Vickers microhardness test. Three indentations were formulated on the samples (n = 10), with a load of 300 gf applied for 15 s to achieve a final average of the evaluations.

-Sorption and solubility

Five of the ten samples tested on the micro-durometer were randomly selected to assess for the sorption and solubility. After microhardness was evaluated, the specimens were kept in a desiccator at 37 ± 2°C. After 24 h, the samples were removed and stored in another desiccator at 23 ± 2°C for 2 h, and then weighed with a precision digital scale (AUW120D, Shimadzu, Kyoto, Japan). This cycle was repeated, with mass loss not exceeding 0.1 mg, until a constant mass was obtained (m1). The diameter and height of each specimen were measured with a caliper to calculate their volume. The samples were immersed in distilled water at 37° C for seven days. After this period, the samples were gently dried with absorbent paper and weighed again (m2).

**Figure 1:**
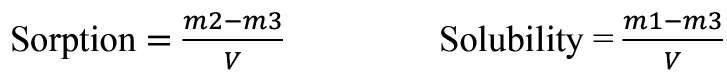
Formula.

After the second weighing, the samples were returned to the desiccator and the process was repeated to obtain a new constant mass (m3). Water sorption and solubility were calculated using the following equations (14), (Fig. 1):

where *m1* is the mass after the sample was initially dried (μg), *m2* is the mass after seven days immersed in water (μg), *m3* is the final mass after the sample was dried (μg), and V is the initial volume of each sample (mm3).

-Color stability

The five samples selected for the evaluation of sorption and solubility and immersed in distilled water alone were used as controls. The others were immersed in a solution of distilled water and coffee, which was prepared by dissolving 0.51 g of instant coffee powder (Nescafé Gold, Rio de Janeiro, Brazil) in 50 ml of distilled water. The immersion solutions were replaced daily.

The samples were evaluated three times: before immersion, after one day of immersion, and after seven days of immersion. The samples were superimposed on a sheet of brown paper with the tip of the digital spectrophotometer (Vita Easy Shade, Wilcos, Rio de Janeiro, Brazil) positioned in the center of each sample. Two measurements were performed at each moment of evaluation to obtain an average.

**Figure 2:**
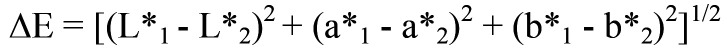
Formula.

The CIE L*a*b* values of the surfaces were measured, and the color difference (ΔE) was obtained from the difference between the evaluations using the following equation (29), (Fig. 2).

where L* refers to brightness, a* to the red-green axis, and b* to the yellow-blue axis. Subscripts 1 and 2 refer to the color coordinates before and after immersion, respectively. A high ΔE value indicates a large color difference.

-Statistical analysis

A bank was built for data analysis on a Microsoft Excel spreadsheet, which was exported to the SPSS software, version 18. The Shapiro-Wilk test was applied to assess the normality of the hardness, sorption, solubility, and color stability scores. In the indication non-normality, the types of composite resin and types of immersion were compared using the Mann-Whitney and Kruskal-Wallis tests, all considering a significance level of 5%.

## Results

Table 2 shows the distribution of microhardness values by group. The FBF group had a higher average (56.38), followed by the OBF (52.49) and TNC (48.43) groups, with a significant difference (*p* <0.001) between the groups.

**Table 2:**
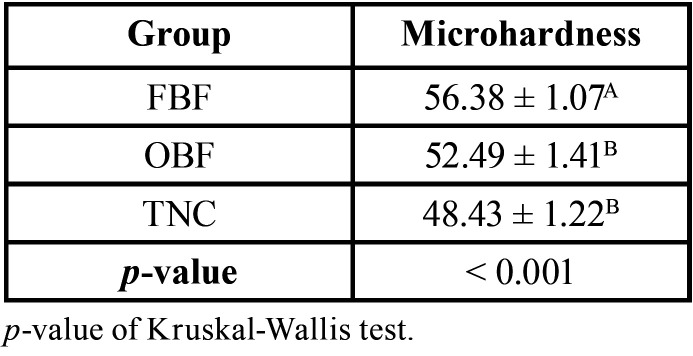
Average and standard deviation of microhardness assessment. Different superscript letters indicate statistical difference.

Table 3 shows the sorption and solubility evaluated in each composite resin group. The highest mean sorption was in the OBF group (16.9 µg / mm3), followed by FBF (16.8 µg / mm3) and TNC (11.3 µg / mm3). There was a significant difference in sorption between groups (*p* = 0.005), indicating that TNC had significantly lower sorption than FBF and OBF.

**Table 3:**
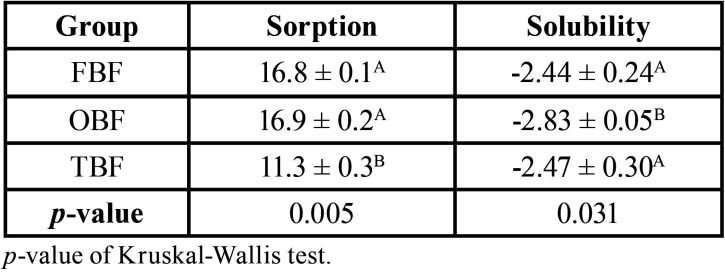
Mean and standard deviation of the sorption and solubility score according to the type of resin.

In the solubility evaluation, the OBF group had a lower mean value (-2.83 µg / mm3). There was a significant difference (*p* = 0.031), indicating that the OBF group had a significantly lower solubility value than the other groups. Negative solubility values indicate mass increase.

Table 4 shows the color stability assessment (∆E) for different groups, times of assessment, and immersion solutions (coffee or water). There was a significant difference in the mean of the ∆E1day score for both immersion solutions in all groups (*p* <0.005), with a higher value for immersion in coffee. Regarding color variation between the groups, there was a significant difference for coffee (*p* = 0.008). The TNC group had a lower mean ∆E score after 1 day of immersion in coffee (9.17 points), followed by OBF (9.48) and FBF (12.62) (*p* = 0.009), indicating that FBF resin showed greater pigmentation potential after 1 day.

**Table 4:**
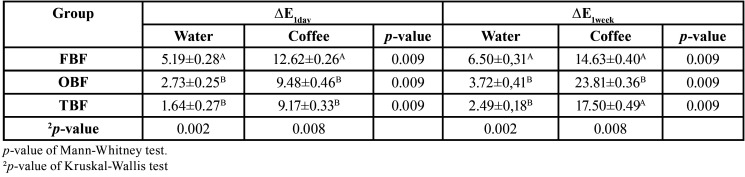
Average and standard deviation of ∆E at 1 day and at 1 week of evaluation, according to the type of resin and the immersion solution.

In the distribution of the ∆E1week score by group and immersion solution, there was a significant difference for immersion in water or coffee in all types of resins evaluated (*p* = 0.009), with a higher value of ∆E when immersed in coffee. When the resin was immersed in coffee, a lower ∆E1week score was found in FBF (14.63), followed by TNC (17.50) and OBF (23.81). Regarding the distribution of the ∆E1week score between the resins two by two, the comparison test was significant for the OBF group compared to the other two (*p* = 0.008). 

## Discussion

All null hypotheses were rejected. The FBF composite resin showed statistically better hardness values than the other groups (*p* <0.001). After 7 d of immersion in coffee, the OBF resin showed statistically inferior color stability results compared to the other groups (*p* = 0.008). Regarding sorption, the TNC resin showed statistically significant results compared to the other groups (*p* = 0.005). OBF showed statistically inferior solubility results compared to other materials (*p* = 0.031).

Due to the popularization of composite resins as a restorative material, many studies have sought improvements for their mechanical and optical properties (16). However, there remain drawbacks inherent to composites, such as incomplete polymeric conversion, water sorption, and stress generated by polymerization contraction (3).

The correct light curing of materials is one of the determining factors in a restoration’s longevity, and it is directly related to the analysis of microhardness (17). Absorption photoinitiators of shorter wavelength appear ineffective in deeper areas (18). This fits with other studies in which the photopolymerization of bulk fill composite resins containing only camphorquinone as a photoinitiator resulted in greater Knoop microhardness for LED monowave lights instead of polywave lights (18).

Tetric N-Ceram Bulk Fill resin contains alternative photoinitiators intended to enhance photopolymerization, such as Ivocerin (derivative dibenzoyl germanium) and TPO (mono aylphosphine oxide), which are stimulated by different wavelengths (19). The presence of these photoinitiators prevented the TNC group from presenting high microhardness values. It was believed that a polywave photopolymerizer could trigger a more effective photopolymerization of this material. However, Gan *et al.* evaluated different bulk fill resins (including Tetric N-Ceram) polymerized with different photopolymerizers (monowave and polywave) and found no statistically significant differences in hardness (20). Another study also found no effect on the hardness of bulk fill composite resins polymerized with either monowave or polywave equipment (21).

In the present study, the FBF and OBF groups with only camphorquinone as a photoinitiator showed higher values of microhardness, as expected. The increase in curing depth may be due to the greater absorption of visible light by this photoinitiator. Photopolymerization was carried out with a monowave LED light in continuous mode with 1200 mW / cm2 of light intensity for 40 s. Several studies have evaluated the capacity of camphorquinone and Ivocerin to be sensitized by the range of light emission used (18,22).

The lower percentage of charge particles in the composition of the TNC group may explain its lower hardness compared to the other materials tested (Table 1). Nascimento *et al.* evaluated the hardness of 9 bulk fill resins and also found a correlation between the fewest filler particles and the lowest hardness (23).

Correct polymerization is a major concern with the use of bulk fill resins. Some studies claim that the polymerization reaction of composite resins can continue for several days (24), and that the degree of conversion can vary from 34.7% to 77.1% and may increase in the first day (24). The composition of the materials, as well as inadequate photopolymerization with low monomeric conversion, can result in greater solubility, as these residual monomers are the main components released when restorations are exposed to the oral environment (25).

In this study, the specimens were kept in a desiccator at 37 ± 2 °C for 24 h; subsequently, these were removed and stored in another desiccator at 23 ± 2 °C for 2 h, and later weighed with a precision digital scale. The initial and final dehydration can directly affect the solubility value of the material. Given that the samples were not completely dehydrated at the beginning of the process, the solubility values may reflect only the end of the desiccation of the samples. The study by Mortier *et al.* evaluated the solubility of different materials with and without the initial dehydration cycle, concluding that there is an increase in solubility of up to 8 times when specimens are not dehydrated (26). The methodology used in the present study follows the norm ISO 4049: 2009 and favors a better comparison between previously established studies.

Composite resins must have a solubility of less than 7.5 µg / mm3 for a storage period of 7 d in order to be safely indicated according to the ISO standard (15). All the materials tested in this study showed values well below this limit (Table 3). Although there is a statistically significant difference, all materials tested were within the satisfactory standards.

The release of composite resin products has been extensively investigated *in vitro* by immersing samples in solutions such as water or organic solvent (27). Usually, an analysis is performed after 24 h or 1 week, with few studies covering longer periods, such as months or even a year (24). In a study by Putzeys *et al.*, monomers were quantified in the samples depending on the composite and the extraction solution, and monomers such as Bis-GMA, HEMA, and UDMA were observed to dissociate from the materials continuously during immersion for up to 52 weeks (28). The materials continued to release small amounts of monomers for longer periods. Even if this monomer dissociation does not represent high toxicity or a short-term risk to human health, it can cause chronic exposure and health risks in the long term, which should not be ignored (28).

For a material to be aesthetic, we must consider not only its optical effects in mimicking natural teeth, but also its ability to retain a sTable color against the challenges of the oral environment. When a composite resin restoration is completed and exposed to the moist oral environment, saliva water is incorporated into it and can generate hydrolytic and hygroscopic effects in different proportions (14). The staining of composite resins is multifactorial, involving intrinsic and extrinsic factors (29).

Extrinsic factors include sorption of dyes from food, which can highlight coffee, soft drinks, alcoholic beverages, teas, juices, and habits such as smoking due to the presence of nicotine (29). In this study, color was measured with a digital spectrophotometer after seven days of immersion in distilled water and coffee. There was a difference between the groups in the 1-day evaluation for both solutions, which was greater when immersed in coffee. This was expected based on findings from other studies (29).

After 1 week of immersion in water, the TNC group had greater color stability (2.49), followed by OBF (3.72) and FBF (6.50), supporting the suggestion that variations in the composition of resins and in the characteristics of the formed polymer may have implications for color stability during storage in water (14,29).

In contrast, after 1 week of immersion in coffee, the FBF group showed better stability (14.63), followed by TNC (17.50) and then OBF (23.81). In a study by Ruyter *et al.*, color change after immersion in a dye solution was calculated by an ΔE equation, with values less than 1 and values greater than 3.3 considered unacceptable (30). Stain intensity can vary based on the type of solution, its pH, frequency, and permanence in contact with the dye.

Clinically, this is associated with poor oral hygiene, presence of biofilm, oxidation of the oral environment, and increased solubility, causing a change in the composite resin’s color (30). A greater amount of filler particles in a composite resin can also increase resistance to staining (29,30). This finding is consistent with the present study, where the group with the highest amount of filler particles (FBF) also showed the greatest color stability after 7 d of immersion in coffee.

## Conclusions

All composite resins tested in this study showed acceptable sorption and solubility results as recommended by ISO 4049-2009. Filtek Bulk Fill resin presented higher microhardness results than the other materials. Among the materials tested, Opus Bulk Fill composite resin pigmented the most after 7 d of immersion in coffee.

## References

[1] Benetti AR, Havndrup-Pedersen C, Honoré D, Pedersen MK, Pallesen U (2015). Bulk-Fill Resin Composites: Polymerization Contraction, Depth of Cure, and Gap Formation. Operative Dentistry.

[2] Alqudaihi FS, Cook NB, Diefenderfer KE, Bottino MC, Platt JA (2019). Comparison of internal adaptation of bulk-fill and increment-fill resin composite materials. Operative dentistry.

[3] Veloso SRM, Lemos CAA, Moraes SLD, Egito Vasconcelos BC, Pellizzer EP, Monteiro GQM (2019). Clinical performance of bulk-fill and conventional resin composite restorations in posterior teeth: a systematic review and meta-analysis. Clinical oral investigations.

[4] Van Ende A, De Munck J, Lise DP, Van Meerbeek B (2017). Bulk-Fill composites: A review of the current literature. J Adhes Dent.

[5] Chesterman J, Jowett A, Gallacher A, Nixon P (2017). Bulk-fill resin-based composite restorative materials: A review. Brazilian Dental Journal.

[6] Ide K, Nakajima M, Hayashi J, Hosaka K, Ikeda M, Shimada Y (2019). Effect of light-curing time on light-cure/post-cure volumetric polymerization shrinkage and regional ultimate tensile strength at different depths of bulk-fill resin composites. Dental materials journal.

[7] Maghaireh GA, Price RB, Abdo N, Taha NA, Alzraikat H (2019). Effect of thickness on light transmission and vickers hardness of five bulk-fill resin-based composites using polywave and single-peak light-emitting diode curing lights. Operative dentistry.

[8] Schroeder T, da Silva PB, Basso GR, Franco MC, Maske TT, Cenci MS (2019). Factors affecting the color stability and staining of esthetic restorations. Odontology.

[9] Bahbishi N, Mzain W, Badeeb B, Nassar HM (2020). Color Stability and Micro-Hardness of Bulk-Fill Composite Materials after Exposure to Common Beverages. Materials.

[10] Oliveira DC, Rocha MG, Gatti A, Correr AB, Ferracane JL, Sinhoret MA (2015). Effect of different photoinitiators and reducing agents on cure efficiency and color stability of resin-based composites using different LED wavelengths. Journal of dentistry.

[11] Shamszadeh S, Sheikh-Al-Eslamian SM, Hasani E, Abrandabadi AN, Panahandeh N (2016). Color stability of the bulk-fill composite resins with different thickness in response to Coffee/Water immersion. International journal of dentistry.

[12] Ardu S, Duc O, Di Bella E, Krejci I, Daher R (2018). Color stability of different composite resins after polishing. Odontology.

[13] Dhananjaya KM, Vadavadagi SV, Almalki SA, Verma T, Arora S, Kumar NN (2019). In Vitro Analysis of Different Polishing Systems on the Color Stability and Surface Roughness of Nanocomposite Resins. The journal of contemporary dental practice.

[14] Brito OFF, Oliveira ILM, Monteiro GQM (2019). Hydrolytic and Biological Degradation of Bulk-fill and Self-adhering Resin Composites. Operative dentistry.

[15] (2009). ISO 4049: Dentistry polymer - based filling, restorative and luting materials.

[16] Barutcigil Ç, Barutcigil K, Özarslan MM, Dündar A, Yilmaz B (2018). Color of bulk-fill composite resin restorative materials. J Esthet Restor Dent.

[17] Shimokawa C, Turbino ML, Giannini M, Braga RR, Price RB (2018). Effect of light curing units on the polymerization of bulk fill resin-based composites. Dental Materials.

[18] Rocha MG, de Oliveira D, Correa IC, Correr-Sobrinho L, Sinhoreti M, Ferracane JL (2017). Light-emitting diode beam profile and spectral output influence on the degree of conversion of bulk fill composites. Operative Dentistry.

[19] Moszner N, Fischer UK, Ganster B, Liska R, Rheinberger V (2008). Benzoyl germanium derivatives as novel visible light photoinitiators for dental materials. Dent Mater.

[20] Gan JK, Yap AU, Cheong JW, Arista N, Tan C (2018). Bulk-fill composites: effectiveness of cure with poly-and monowave curing lights and modes. Operative dentistry.

[21] Menees TS, Lin CP, Kojic DD, Burgess JO, Lawson NC (2015). Depth of cure of bulk fill composites with monowave and polywave curing lights. American journal of dentistry.

[22] Rodriguez A, Yaman P, Dennison J, Garcia D (2017). Effect of Light-Curing Exposure Time, Shade, and Thickness on the Depth of Cure of Bulk Fill Composites. Operative Dentistry.

[23] Nascimento AS, Lima DB, Fook MVL, Albuquerque MS, Lima EA, Sabino MA (2018). Physicomechanical characterization and biological evaluation of bulk-fill composite resin. Brazilian oral research.

[24] Alshali RZ, Silikas N, Satterthwaite JD (2013). Degree of conversion of bulk fill compared to conventional resin-composites at two times intervals. Dent. Mater.

[25] Okada K, Tosaki S, Hirota K, Hume WR (2001). Surface Hardness change of restorative filing marerials stored in saliva. Dent Mater.

[26] Mortier E, Gerdolle DA, Dahoun A, Panighi MM (2005). Influence of initial water content on the subsequent water sorption and solubility behavior in restorative polymers. Am J Dent.

[27] Van Landuyt KL, Nawrot T, Geebelen B, De Munck J, Snauwaert J, Yoshihara K (2011). How much do resin-based materials release? A meta-analytical approach. Dent Mater.

[28] Putzeys E, Nys S, Cokic SM, Duca RC, Vanoirbeek J, Godderis L (2019). Long-term elution of monomers from resin-based dental composites. Dent Mater.

[29] Shiozawa M, Takahashi H, Asakawa Y, Iwasaki N (2015). Color stability of adhesive resin cements after immersion in coffee. Clinical oral investigations.

[30] Ruyter IE, Nilner K, Moller B (1987). Color stability of dental composite resin materials for crown and bridge veneers. Dent Mater.

